# Global Whole-Genome Resequencing of Beef Cattle Reveals Characteristic Traits Related Genes in Pinan Cattle

**DOI:** 10.3390/ani15111626

**Published:** 2025-05-31

**Authors:** Dongdong Bo, Yuanyuan Wang, Yilin Bai, Jing Li, Jiameng Shen, Jinxiao Wei, Yueyu Bai

**Affiliations:** 1 School of Agricultural Sciences, Zhengzhou University, Zhengzhou 450001, China; dongdbo@foxmail.com (D.B.); wyshll2022@163.com (Y.W.); bylin213@zzu.edu.cn (Y.B.); chnlijing@zzu.edu.cn (J.L.); jmengshen@163.com (J.S.); 2 Key Laboratory of Innovative Utilization of Local Cattle and Sheep Germplasm Resources (Co-Construction by Ministry and Province), Ministry of Agriculture and Rural Affairs, Zhengzhou 450001, China; 3 Henan Provincial Center of Seed Industry Development, Zhengzhou 450007, China; 13523095527@163.com; 4 Institute of Livestock Breeding and Reproduction, Henan University, Zhengzhou 450046, China

**Keywords:** Pinan cattle, selection signature, candidate genes, population genetic structure, *NDN* gene, *PARVA* gene

## Abstract

Beef cattle breed improvement holds strategic significance in the livestock industry. Pinan cattle, developed through years of selective breeding in Xinye County, Henan Province, exhibit superior traits including thin skin, fine bone structure, rapid growth, high dressing percentage, excellent meat yield, and superior feed efficiency. However, research on the genetic characteristics of Pinan cattle remains in its infancy. In this study, we investigated population genetic diversity and positive selection signals in Pinang cattle based on whole-genome resequencing data. Using a selective sweep approach, we identified 98 candidate genes associated with growth, reproduction, and immunity, along with 13 high-confidence missense mutations, which may underlie key traits in this population. Based on the critical roles of the *NDN* and *PARVA* genes in reproduction and muscle development, the predominant T allele at the *NDN* c.581T > A and *PARVA* c.893T > A loci in the Pinan cattle population may partially explain their advantages in sexual precocity and rapid growth compared to other breeds or populations. This study provides an important theoretical basis for the genetic improvement of native beef cattle and lays a scientific foundation for further investigation into the growth and development mechanisms of Pinan cattle.

## Simple Summary

Whole-genome resequencing has been increasingly applied to beef cattle genetic breeding. As a superior indigenous breed in Henan Province, Pinan cattle exhibit rapid growth, early sexual maturity, and high dressing percentage, making them an important component of Chinese local beef cattle genetic resources. However, their genomic variation remains poorly characterized. Here, we performed whole-genome resequencing on Pinan cattle to assess genetic diversity and conducted selective sweep analysis to identify candidate genes and loci linked to key economic traits. This study provides insights into the molecular basis of Pinan cattle’s superior traits and supports marker-assisted breeding for growth and reproductive performance.

## 1. Introduction

The beef cattle industry holds an important position in the Chinese livestock sector, with beef production significantly influencing meat market dynamics [1]. Within the global agricultural resource system, cattle genetic resources have become increasingly prominent as vital livestock assets. They serve not only as key components for meeting premium meat market demands but also as driving forces for optimizing agricultural industrial structures and stimulating rural economic development [2,3]. In livestock advancement, superior genetics leads the way; the breeding industry, being fundamental to agricultural modernization, is indisputably vital for sustainable beef production systems.

The Pinan cattle, as an independent beef breed, were developed using Nanyang cattle as the maternal parent and introducing Italian Piedmont cattle as the paternal parent, through hybrid innovation, cross fixation, and generational breeding (with 75% external blood) [4]. As a key genetic resource for the Chinese beef industry, it exhibits superior growth and economic traits. Under identical conditions, it reaches market weight by 18 months, 6 months earlier than Nanyang yellow cattle [5]. Its slaughter performance is outstanding, with a 65.2% dressing percentage and 55.6% meat yield (10% higher than the dam breed), while carcass meat yield remains stable at 84.2–85.9% [6,7]. Notably, it shows early sexual maturity (first estrus at 9 months, breeding age at 12 months, 3 months earlier than Nanyang cattle), outperforming most beef breeds [8]. These advantages (rapid growth, high meat yield, feed efficiency, and early maturity) solidify its role as a vital genetic resource.

In-depth exploration of the genes behind the excellent traits of Pinan cattle and their molecular regulatory mechanisms will help accelerate the breeding process [9]. However, genomic-level studies of growth-related genetic variations in Pinan cattle remain limited. This study analyzed the genetic diversity of different beef cattle populations using whole-genome resequencing data from 352 cattle, including 10 Pinan cattle. We identified positively selected genes and loci in Pinan cattle through selective sweep analysis that may influence their economically important traits. We aim to reveal key genomic signatures underlying the breed’s characteristic traits by mapping these selective sweep regions. These findings provide valuable insights into the genetic basis of Pinan cattle’s distinctive characteristics and offer scientific support for population expansion and breeding programs.

## 2. Materials and Methods

### 2.1. Ethics Statement

The study was conducted in strict compliance with the code of animal ethics (Zhengzhou University Ethics Approval Number: ZZUIRB2024-102, Approval Date: 4 February 2024). Blood samples were collected by certified veterinarians in accordance with the Zhengzhou University Animal Welfare Protocol. All methods were performed in accordance with relevant guidelines and regulations.

### 2.2. Sample Preparation

**Figure 1:**
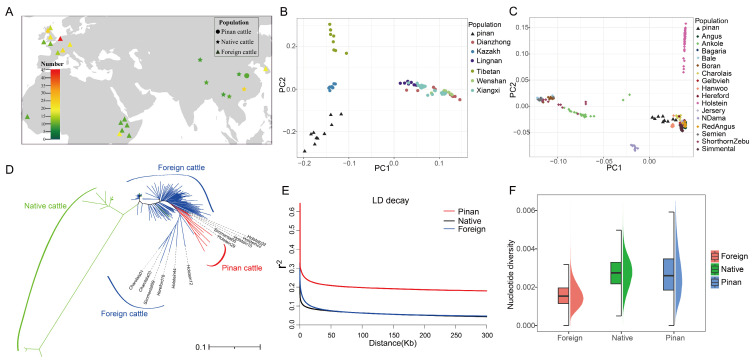
Genetic structure and diversity of Pinan cattle. (**A**) Geographic distribution map of populations. (**B**) Principal component analysis (PCA) of Pinan cattle and native cattle. (**C**) Principal component analysis of Pinan cattle and foreign cattle. (**D**) Neighbor-joining (NJ) tree of 352 individuals. The red, green, and blue colors represent the Pinan cattle population, the native cattle population, and the foreign cattle population, respectively. (**E**) Linkage disequilibrium (LD) decay patterns across three populations, with colored lines representing distinct groups. (**F**) Nucleotide diversity (*π*) among populations, with midline values indicated by black lines in boxplots.

Whole-genome resequencing was performed on blood samples collected from ten Pinan cattle in Xinye County, Henan Province. Additionally, to better analyze the genetic diversity of Pinan cattle, this study incorporated an additional 342 resequenced samples with wide geographical distributions, encompassing six native breeds [Kazakh (*n* = 9), Tibetan (*n* = 9), Lingnan (*n* = 8), Wenshan (*n* = 8), Dianzhong (*n* = 10), and Xiangxi (*n* = 23)] and sixteen foreign breeds [Angus (*n* = 25), RedAngus (*n* = 16), Hereford (*n* = 21), Jersery (*n* = 12), Holstein (*n* = 45), Charolais (*n* = 14), Gelbvieh (*n* = 21), Simmental (*n* = 23), Hanwoo (*n* = 18), ShorthornZebu (*n* = 10), Ankole (*n* = 20), Boran (*n* = 10), Ndama (*n* = 10), Bagaria (*n* = 10), Semien (*n* = 10), and Bale (*n* = 10)] (Table S1, Figure 1A).

### 2.3. Read Mapping and SNP Calling

Raw sequencing data were processed using Trimmomatic [10] (v0.39) for quality filtering, followed by quality control assessment with FastQC [11] (v0.12.1). The clean reads were aligned to the reference genome (ARS-UCD1.2) by employing the BWA tool [12] (v0.7.17). Sequencing depth and coverage were calculated using SAMtools [13] (v1.16.1). After removing potential PCR duplicates with Picard tools, variant calling was performed through the GATK [14] (v4.1.0.0) pipeline, including the following modules: HaplotypeCaller for initial variant detection, CombineGVCFs for joint genotyping, GenotypeGVCFs for variant consolidation, and VariantFiltration for quality filtering with the parameters: “DP < 10, QD < 2.0, FS > 60.0, SOR > 4.0, MQ < 40.0, MQRankSum < −12.5, ReadPosRankSum < −8.0”. Finally, SNPs were filtered using VCFtools [15] (v0.1.16).

### 2.4. Population Genetic Analysis

We used Plink to calculate genotype frequencies and performed principal component analysis (PCA) to extract PC1 and PC2 principal components. The ggplot2 package of the R language (v4.3.1) was utilized to plot 2D scatterplots to demonstrate population clustering. Mitochondrial genomic variation data were extracted by Vcftools (v0.1.16) and combined with VCF2D to calculate the genetic distance matrix. Based on this matrix, a phylogenetic tree was constructed using the neighbor-joining (NJ) method of MEGA12 and optimized for visualization using the iTOL online platform (https://itol.embl.de/, accessed on 20 March 2025). To further characterize genomic features across populations, we evaluated linkage disequilibrium (LD) decay patterns using PopLDdecay [16] (v3.42) by calculating allele frequency correlations (*r*^2^). Moreover, we calculated the genome-wide nucleotide diversity (*π*) through VCFtools. The calculation was performed with a sliding window size of 100 kb and a step size of 10 kb.

### 2.5. Detection of Selective Signatures

Genome-wide selective signatures were detected using a multidimensional population genetics approach. Genetic differentiation between populations was assessed by calculating the fixation index (*F_ST_*) in VCFtools (v0.1.16) with 100-kb sliding windows and 10-kb step sizes. The top 5% of *F_ST_* values (native cattle vs. Pinan cattle) and *F_ST_* values (foreign cattle vs. Pinan cattle) were selected as candidate genomic regions under selection. Additionally, we computed the *θπ* ratio (*θπ*_native/Pinan_ and *θπ*_foreign/Pinan_) and identified the top 5% of windows as putative positively selected regions. Combined with *Tajima’s D* neutrality test, windows with *Tajima’s D* values less than 0 were selected as putative selected regions. Finally, using the intersect module in Bedtools [17] (v 2.17.0), we retained only the overlapping regions detected by all three methods as high-confidence selective sweeps in Pinan cattle.

### 2.6. Enrichment Analysis

The Database for Annotation, Visualization, and Integrated Discovery (DAVID [18], https://david.ncifcrf.gov/home.jsp, accessed on 26 December 2024) was employed to conduct gene set enrichment analysis for GO terms and KEGG pathways. Significance was attributed solely to GO terms and KEGG pathways exhibiting a *p*-value of less than 0.01.

### 2.7. Protein Structure and Function Prediction

The amino acid sequences of the wild-type and mutant proteins were submitted to SWISS-MODEL [19] (https://swissmodel.expasy.org/, accessed on 14 February 2025) for structural modeling. The highest-scoring model from each prediction was selected based on comprehensive evaluation scores and downloaded in PDB format for subsequent structural visualization using PyMOL [20]. The physicochemical properties of the proteins were predicted using ProtParam (https://web.expasy.org/protparam/, accessed on 16 February 2025). Hydropathy analysis was performed using ProtScale (https://web.expasy.org/protscale/, accessed on 16 February 2025) to evaluate the impact of mutations on the local hydrophobicity profiles of the encoded proteins. The potential effect of the mutation on conserved protein domains was evaluated using the Conserved Domain Database (CDD) [21] (https://www.ncbi.nlm.nih.gov/Structure/cdd/cdd.shtml, accessed on 18 February 2025). Finally, the functional impact of the mutation was predicted using SIFT [22] (https://sift.bii.a-star.edu.sg/www/SIFT_seq_submit2.html, accessed on 16 February 2025), which estimates whether an amino acid substitution affects protein function.

## 3. Results

### 3.1. Data Analysis and SNP Calling

This study analyzed a whole-genome resequencing dataset comprising 352 cattle (Table S1), with an average mapping rate and sequencing depth of 99.06% and 15.43×, respectively (Table S2). We identified 90,854,225 single-nucleotide polymorphisms (SNPs) randomly distributed across autosomes, yielding an overall SNP density of 3.69% (i.e., 3.69% of autosomal loci were polymorphic). Transition mutations predominated (n = 62,767,336), accounting for 69.9% of total SNPs. The 352 cattle were categorized into three groups—Pinan cattle, native cattle, and foreign cattle—for subsequent analysis.

### 3.2. Population Genetic Structure and Diversity Analysis

Based on genomic SNP data, we analyzed the genetic structure and diversity of Pinan cattle and other cattle populations using a combination of PCA, NJ phylogenetic tree construction, LD decay analysis, and *π* computation (Figure 1). Pinan cattle were genetically clustered closer to European and Northeast Asian cattle breeds (Angus, Charolais, Gelbvieh, Hanwoo, Hereford, Jersery, RedAngus, and Simmental) (Figure 1B,C), and phylogenetic analyses further showed that they were more closely related to the European cattle groups (Simmental, Holstein, and Charolais) (Figure 1D and Figure S1). Genetic characterization revealed that, compared to both native and foreign cattle populations, the Pinan cattle population exhibited the slowest LD decay rate, with an approximate 50 kb distance when *r*^2^ reached 0.20, the longest among the three groups (Figure 1A). The Pinan population showed comparable genetic diversity levels to native cattle, both being higher than foreign breeds (Figure 1B). This genetic diversity pattern suggests that Pinan cattle have effectively maintained the genetic characteristics of local breeds during selective breeding, while simultaneously demonstrating considerable potential for genetic improvement.

### 3.3. Genetic Signature of Positive Selection in Pinan Cattle

**Figure 2:**
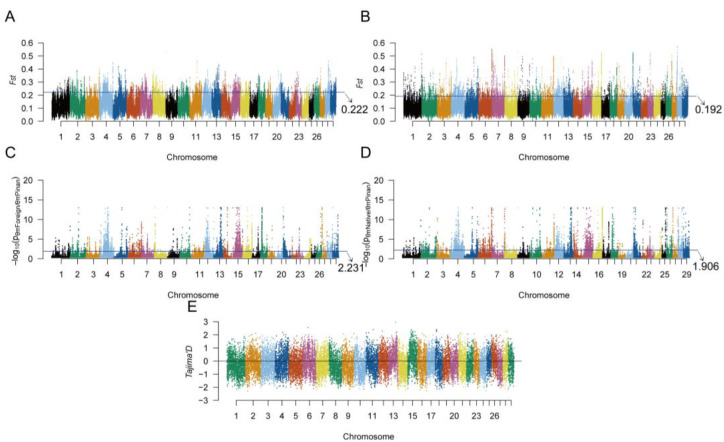
Positive selection signatures in Pinan cattle. (**A**,**B**) Manhattan plots showing on each chromosome *F_ST_* distributions between Pinan cattle and (**A**) native cattle or (**B**) foreign cattle populations. The blue horizontal lines indicate the 95th percentile thresholds (top 5%) for *F_ST_* values, highlighting putative selective sweeps. (**C**,**D**) Significance profiles of *θπ* ratios (Pinan cattle vs. native cattle in (**C**); Pinan cattle vs. foreign cattle in (**D**)). Z-score normalized *θπ* ratios were used to determine statistical significance, with genomic regions above the blue line (top 5% of *p*-values) considered under selection. (**E**) Chromosomal distribution of *Tajima’s D* values in Pinan cattle. Genomic windows with significantly negative values (*D* < 0) were identified as putative selected regions.

We employed complementary approaches to detect selection signatures: population differentiation was assessed via fixation index (*F_ST_*) calculations; nucleotide diversity was analyzed through *π* (Pi) statistics; and neutrality was evaluated using *Tajima’s D* tests. For each method, candidate regions were identified as the top 5% of *F_ST_* and *π* values, and genomic windows with *Tajima’s D* < 0. High-confidence positive selection regions in Pinan cattle were subsequently defined as overlapping intervals consistently detected by all three methods (Figure 2).

**Figure 3:**
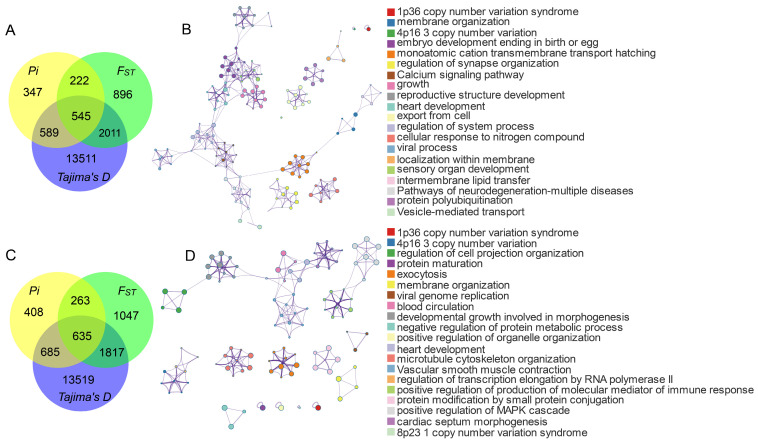
Functional enrichment analysis of positively selected genes in Pinan cattle. (**A**,**B**) Positive selection genes and their GO and KEGG results in the comparison of Pinan cattle with native cattle by the three methods, respectively. (**C**,**D**) Positively selected genes and their GO and KEGG results in the comparison of Pinan cattle with foreign cattle by the three methods, respectively.

Genome-wide selective sweep analysis of Pinan cattle identified 545 and 635 high-confidence positively selected genes (PSGs) when compared with native cattle and foreign cattle breeds, respectively (Figure 3). Functional enrichment analysis revealed significant enrichment of these genes in multiple biological processes and pathways associated with growth, reproduction, and immune functions. Terms related to growth, reproduction, and immunization include embryo development ending in birth or egg hatching (GO:0009792), growth (GO:0040007), reproductive structure development (GO:0048608), heart development (GO:0007507), sensory organ development (GO:0007423), intermembrane lipid transfer (GO:0120009), developmental growth involved in morphogenesis (GO:0060560), and positive regulation of production of molecular mediators of immune response (GO:0002702). Based on GO and KEGG pathway enrichment analyses, we identified 98 positively selected genes (Table S3, Figure 3) associated with growth, reproduction, and immunity from the candidate regions.

### 3.4. Consequences of Positively Selected SNPs on Protein

**Figure 4:**
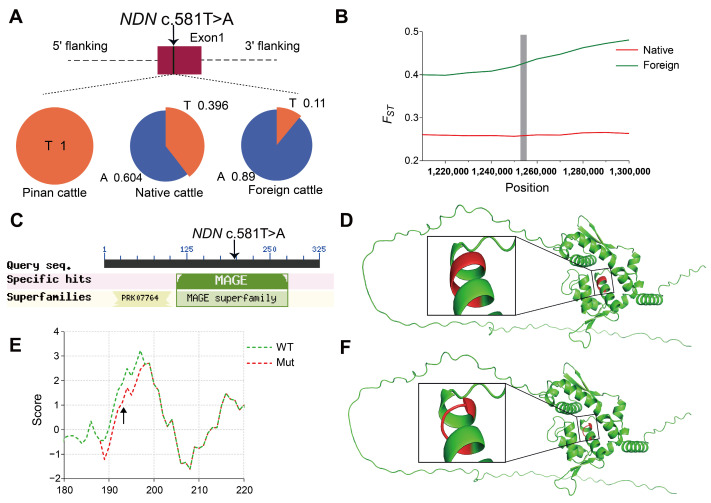
The effect of positively selected locus *NDN* c.581T > A on NDN protein structure and function. (**A**) The location of *NDN* c.581T > A and the allele frequency across three populations. (**B**) *F_ST_* values for the *NDN* c.581T > A locus and nearby loci in the *NDN* gene, with the gray bar indicating the area around *NDN* c.581T > A. (**C**) Conserved domains of *NDN*, with the *NDN* c.581T > A variant marked (arrow). (**D**,**F**) Predicted structures of (**D**) WT and (**F**) Mut NDN proteins. The red regions indicate the mutation site and surrounding sites, and the boxed area highlights the α-helix altered by *NDN* c.581T > A. (**E**) Hydrophobicity profiles of wild-type (WT, green dashed line) and mutant (Mut, red dashed line) NDN proteins. The arrow indicates the residue altered by NDN c.581T > A (p.Gly194His).

**Figure 5:**
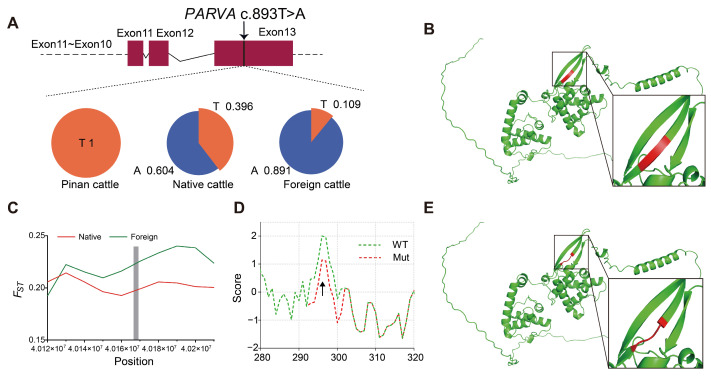
The effect of positively selected locus *PARVA* c.893T > A on PARVA protein structure and function. (**A**) The location of *PARVA* c.893T > A and the allele frequency across three populations. (**B**,**E**) Predicted structures of (**B**) WT and (**E**) Mut PARVA proteins. The red regions indicate the mutation site and surrounding sites, and the boxed area highlights the α-helix altered by *PARVA* c.893T > A. (**C**) *F_ST_* values for the *PARVA* c.893T > A locus and nearby loci. The gray bar indicates the area around *PARVA* c.893T > A. (**D**) The hydrophobicity profiles of wild-type (WT, green dashed line) and mutant (Mut, red dashed line) PARVA proteins. The arrow indicates the residue altered by *PARVA* c.893T > A (p.Val298Glu).

Through comparative analysis of allele frequency differences between Pinan cattle and both native and foreign cattle populations, we identified 13 high-confidence missense variants under positive selection in Pinan cattle (Table 1). These variants are located in key functional domains of genes associated with growth, reproduction, and immune response, suggesting potential effects on encoded protein structure and function that may contribute to phenotypic traits in Pinan cattle. Functional analyses demonstrated that these missense mutations significantly altered encoded protein characteristics (Table S4, Figure 4 and Figure 5). Notably, the *NDN* c.581T > A (p.Gly194His) and the *PARVA* c893T > A (p.Val298Glu) were both found to have a large effect on the structure of their encoded proteins.

#### 3.4.1. Effects of NDN c.581T > A on NDN Protein Structure and Function

Necdin (*NDN*) belongs to the melanoma-associated antigen (MAGE) family, exhibiting tissue-specific expression in male germ cells, placental tissues, and embryonic development-associated cells. The genomic region containing *NDN* co-localizes with quantitative trait loci (QTLs) associated with bovine fertility and early growth traits [23,24]. This study identified significant differences in allele frequencies of the *NDN* c.581T > A locus (located in exon 13th) between Pinan cattle and two others (Figure 4A). Furthermore, this locus is within a conserved MAGE homology domain (Figure 4C), which plays crucial roles in tumorigenesis and reproductive development. Our selective sweep analysis identified strong signatures of positive selection at this locus (Figure 4B). Additionally, this mutation may enhance the hydrophilicity of the encoded amino acid and adjacent residues (Figure 4E), thereby affecting the physicochemical properties of the protein. Comparative structural analysis reveals that the mutant NDN protein lacks a critical α-helical domain present in the wild-type protein (Figure 4D,F). As α-helices are essential for maintaining protein structure and mediating molecular interactions, this loss is likely to impair *NDN*’s functional integrity. These findings provide new molecular evidence for elucidating the role of the *NDN* gene in the reproduction and growth development of Pinan cattle.

#### 3.4.2. Effects of PARVA c.893T > A on PARVA Protein Structure and Function

Positive selection signals were also identified at *PARVA* c.893T > A in Pinan cattle (Figure 5C), which showed significantly divergent allele frequencies from both native and foreign cattle breeds (Figure 5A). Further analysis revealed that this mutation enhances the hydrophilicity of the encoded amino acid (Figure 5D) and results in the disappearance of the β-sheet structure of the protein (Figure 5B,E). As a member of the parvin family of cytoskeletal proteins, the *PARVA* gene encodes an actin-binding protein. This protein is essential for cell adhesion, migration, and signal transduction, and is closely associated with muscle tissue differentiation. Structural changes in the *PARVA* gene may be involved in regulating important phenotypes, such as muscle development in Pinang cattle, by affecting protein interactions.

## 4. Discussion

Pinan cattle, originating from Xinye County, Henan Province, China, exhibit superior meat production characteristics [25,26]. However, the candidate genes associated with their characteristic traits remain to be fully identified, and research on their genetic features is still in its preliminary stages [27,28]. Therefore, we performed whole-genome resequencing of Pinan cattle and investigated the genetic diversity and genomic regions under selection in Pinan cattle using genome resequencing data from other cattle populations worldwide.

### 4.1. Discussion on the Population Genetic Structure and Diversity Analysis

We analyzed the population genetic structure and diversity of Pinan cattle within the context of global bovine populations. The NJ phylogenetic tree and PCA results showed that Pinan cattle are closely related to European cattle populations (Simmental, Charolais, etc.). Genetic diversity reflects variations in allele frequencies and distributions within a population [29,30]. LD decay analysis revealed a slower decay rate in Pinan cattle, suggesting this population may have experienced strong genetic drift or selection pressure, resulting in closer physical proximity and higher linkage between variant loci. Furthermore, the observed slower LD decay rate may also be attributed to a smaller effective population size, as reduced population size decreases recombination events, thereby slowing LD decay [31,32]. Nucleotide diversity analysis revealed that Pinan cattle exhibit genetic diversity levels comparable to native cattle and significantly higher than foreign bovine populations. This pattern may originate from low-recombination-rate regions in the Pinan cattle genome, which maintain elevated nucleotide diversity while exhibiting slower LD decay. Nearly 30 years of artificial selective breeding in the Pinan cattle population may be responsible for the high LD and lower nucleotide diversity of genomic traits. Collectively, these findings demonstrate that Pinan cattle retain genetic diversity characteristics similar to foreign breeds while displaying distinct genomic features from native cattle through long-term selective breeding, potentially providing the genetic basis for their unique phenotypic traits.

### 4.2. Signature of Selection Analysis

As a high-quality beef cattle germplasm resource, the core advantage of Pinan cattle is reflected in the characteristics of thin skin and fine bone, fast growth, early maturity, high meat yield, and tolerance of roughage. These traits may be linked to candidate genes related to growth regulation, reproductive development, and immune response [9,28,33]. These genes collectively regulate muscle growth and metabolic efficiency through synergistic effects, while simultaneously influencing body conformation development, thereby significantly enhancing overall production performance. While the limited sample size may have reduced detection sensitivity for weaker selective signals or low-frequency variants, the core selection signals remained robust through cross-validation with multiple analytical approaches (*F_ST_*/*π*/*Tajima’s D*). In this study, using a selective sweep approach, we identified 98 candidate genes associated with growth, reproduction, and immune functions in Pinan cattle (Table S3).

Growth-related candidate genes include *POU1F1*, *LGR6*, *FGF10*, *CPNE1, CDON*, and others. The POU class 1 homeobox 1 (*POU1F1*) serves as a crucial transcriptional regulator that plays a vital role in bovine embryonic development and growth regulation. Studies have identified a HinfI polymorphic site in exons 5 and 6 of the bovine *POU1F1* gene, exhibiting three genotypes: AA, BB, and AB [34]. Extensive research has demonstrated that the B allele represents the advantageous allele, with individuals carrying the B allele consistently exhibiting superior growth and development traits compared to those with the A allele. For instance, Di Stasio et al. [35] reported that Piedmontese cattle carrying the B allele showed better meat production performance and growth rate than those with the A allele. Furthermore, in Qinchuan cattle and Jiaxian Red cattle, individuals with the BB genotype displayed significantly greater body weight and body measurements than those with AA or AB genotypes [36,37]. This suggests that the *POUIF1* gene, as a crucial transcriptional regulator, may play an important role in the embryonic development and growth of Pinan cattle. *LGR6* is a member of the leucine-rich repeat-containing G protein-coupled receptor (LGR) family. *Lgr6*-positive cells are essential for proper bone formation through endochondral ossification and significantly contribute to fracture repair processes [38,39]. As a downstream target gene of Wnt/β-catenin signaling, *Lgr6* is essential for fingertip regeneration [40]. Fibroblast growth factor 10 (*FGF10*) plays a critical role in regulating multi-organ developmental processes and maintaining tissue homeostasis. Nurgulsim et al. [41] have identified genotypes GG (g.78), TC (g.116), and TT (g.201) of *FGF10* as significantly correlated with growth and carcass quality traits in Qinchuan cattle. This result enhances the reliability of *FGF10* as a candidate gene affecting growth traits in Pinan cattle. Copine 1, encoded by the *CPNE1* gene, is a calcium-dependent soluble membrane-bound protein that is mainly expressed in skeletal muscle. It has been found that it regulates myoblast proliferation and differentiation through the PERK-eIF2α signaling pathway and is involved in the regulation of muscle production [42,43]. Combined with the results of this study, we suggest that *CPNE1* may be a potential functional gene affecting muscle growth in Pinan cattle. As a co-receptor of the HH signaling pathway, cell adhesion-associated oncogene regulation (*CDON*) regulates motor neuron differentiation, mediates muscle precursor cell interactions, and promotes myogenesis during embryogenesis [44,45]. Furthermore, nuclear-localized DVL1 protein, as a core component of the WNT pathway, is essential for the efficient proliferation of myoblasts [46].

Reproduction-related candidate genes include *DMRT1*, *HOX* family members, *FMN1*, *GATA4*, *PPP3CB*, and others. The doublesex-mab-3-related transcription factor 1 (*DMRT1*) is exclusively found in vertebrates [47]. During early vertebrate evolution, *DMRT1* primarily functions in the germ cell lineage. With the evolution of jawed vertebrates, *DMRT1* regulates somatic masculinization and its maintenance in undifferentiated gonads and testes [48,49]. As a reproduction-related gene, studies of the *DMRT1* gene have focused on humans [50] and mice [51], whereas its function in cattle has not been fully investigated, and it is an important candidate gene. Members of the HOX gene family (including *HOXA2/5-7/9-11/13* and *HOXD13*) are involved in the regulation of embryonic development through the orchestration of limb development, reproductive system differentiation, and organ morphogenesis [52,53]. The *HOXA10* gene may regulate cholesterol synthesis in endometrial stromal cells, and its downregulation can lead to infertility associated with endometrial disorders [54,55]. *HOXA11* is an essential gene for uterosacral ligament (USL) development and is closely associated with female reproductive health and fertility [56,57]. Formin 1 (*FMN1*) belongs to the formin protein family, encodes proteins that dynamically regulate the cytoskeleton. In mammalian cells, *FMN1* rapidly generates long actin microfilaments, contributing to sertoli cell motility, endocytic vesicle-mediated transport, and scaffolding functions [58]. *FMN1* maintains the microtubule and actin skeleton and facilitates sperm transport in the seminiferous tubules [59]. In addition, SNP rs134100268 in the intronic region of the *FMN1* gene was found to be identified as a potential functional locus affecting age at first calving in Canchim beef cattle [60]. It is suggested that the *FMN1* gene may serve as an important candidate gene for reproductive traits in Pinan cattle. The GATA binding protein 4 (*GATA4*), as a core member of the zinc-finger transcription factor GATA family, plays a pivotal regulatory role in reproductive system development and functional maintenance. This transcription factor participates in reproductive regulation through the following mechanisms: regulating cell cycle progression and differentiation modulators in ovarian granulosa cells [61], involvement in molecular regulatory networks controlling germ cell function [62], and serving a decisive role during gonadal differentiation [63]. The *PPP3CB* gene encodes the protein phosphatase 3 catalytic subunit and has not been reported to be associated with traits in Pinan cattle. However, its homologous gene *PPP3CA* affects reproductive traits (e.g., litter size and semen quality) in goats [64]. Therefore, the role of *PPP3CB* in Pinan cattle deserves further investigation.

Immunity-related candidate genes include *IL10*, *NSD2*, *KLK5*, *NOD1*, *SLAMF1*, *TRIM67*, and others. Interleukin-10 (*IL-10*), a common anti-inflammatory cytokine, is produced by various immune cells [65,66]. Widely overexpressed in diverse malignancies, *NSD2* (nuclear receptor binding SET domain protein 2) orchestrates tissue-level immunosuppression and promotes the formation of immunosuppressive niches in tumor microenvironments [67,68]. The kallikrein-related peptidase 5 (*KLK5*) gene, a member of the kallikrein subfamily, is associated with carcinogenesis. Aberrant *KLK5* expression has been identified in cervical cancer and significantly impacts tumor prognosis [69]. Nucleotide-binding oligomerization domain-containing protein 1 (*NOD1*), a member of the NOD-like receptor (NLR) family, represents an important class of intracellular pattern recognition receptors (PRRs) that function in innate immunity. *NOD1* promotes the formation of an antiviral immune environment by increasing IL-8+ cell populations through its involvement in NF-κB and interferon response pathways [70]. Signaling lymphocytic activation molecule family member 1 (*SLAMF1*) modulates rheumatoid arthritis pathogenesis by orchestrating inflammatory cascades via infiltrating immune cells [71]. *SLAMF1*-derived peptides inhibit TLR4-mediated expression of IFNβ and pro-inflammatory factors (TNF, IL-1β, and IL-6) and attenuate LPS-induced lethal shock [72]. Tripartite motif-containing protein 67 (*TRIM67*), an E3 ubiquitin ligase of the TRIM family, orchestrates *DLK1* (delta-like non-canonical Notch ligand 1) ubiquitination via its RING domain, thereby activating Notch signaling to drive *NSCLC* (non-small cell lung cancer) proliferation. These findings position *TRIM67* as a promising therapeutic target in lung cancer [73,74].

### 4.3. Positively Selected Loci NDN c.581T > A and PARVA c.893T > A in Pinan Cattle

In this study, we identified 13 positively selected loci in the Pinan cattle population (Table 1). Notably, two loci, the *NDN* c.581T > A (p.Gly194His) and *PARVA* c.893T > A (p.Val298Glu), showed significant selection signatures, as well as enhanced hydrophilicity of the encoded amino acids and their neighboring residues. This enhanced hydrophilic region may facilitate protein-water interactions, potentially altering their subcellular localization. Furthermore, protein structure prediction analyses confirmed that both variants induce conformational changes in their respective protein secondary structures (Figure 4 and Figure 5).

As a member of the *MAGE* gene family, *NDN* exhibits a strict male germ cell-specific expression pattern in normal tissues and plays critical regulatory roles in mammalian reproductive system development and tumorigenesis [75]. We identified that the *NDN* c.581T > A mutation results in a histidine (His) to glycine (Gly) substitution at position 194, which localizes within the MAGE domain of the NDN protein. The protein structure prediction analysis revealed that the mutation site is located in the MAGE structural domain of NDN protein (Pfam: PF01454), which may severely affect the biological activity of NDN protein, and consequently affect the core biological processes such as germ cell proliferation, differentiation, and meiosis in the organism [76]. The *PARVA* gene encodes a core component of focal adhesions—the cytoskeletal adaptor protein α-parvin, which couples integrins to the actin cytoskeleton via its CH1 and CH2 domains, playing pivotal regulatory roles in cell adhesion and muscle development [77,78]. We showed that the *PARVA* c.893T > A mutation occurs in the hinge region of the CH1 and CH2 structural domains, and that the mutation disrupts the β-sheet structure of the protein. This structural alteration may impair its muscle development-related functions by perturbing the conformational dynamics of the protein.

In summary, the positively selected loci *NDN* c.581T > A and *PARVA* c.893T > A in the Pinan cattle population may collectively constitute the genetic basis for the early-maturity and rapid-growth traits and could serve as key candidate markers for molecular-assisted breeding in this breed. Further functional experimental validation is needed in the future.

## 5. Conclusions

This study investigated the genetic diversity of Pinan cattle using whole-genome resequencing data from 352 individuals. Through selective sweep analysis, we identified a series of positively selected genes and SNPs associated with growth, reproduction, and immunity in this breed. Notably, the selected loci *NDN* c.581T > A and *PARVA* c.893T > A might affect protein function by altering critical domain conformations. Both loci exhibit high T-allele frequencies in Pinan cattle, a genetic signature that may be closely associated with the breed’s superior economic traits, including precocious puberty and rapid growth. These findings provide novel insights into the molecular genetic mechanisms underlying important economic traits in Pinan cattle, while also offering valuable candidate markers for future molecular-assisted breeding programs.

## Figures and Tables

**Table 1:** Positively selected missense variants.

Missense Mutation	Chromosome	ΔAF_1_ ^1^	ΔAF_2_ ^1^	Amino Acid Variation
*DMRT1* c.101G > C	Chr8	0.6212	0.8905	G/A
*DMRT1* c.634T > A	Chr8	0.5791	0.7759	Y/N
*DMRT1* c.881C > G	Chr8	0.5970	0.8905	T/S
*FMN1* c.58G > C	Chr10	0.6045	0.8869	E/Q
*FMN1* c.745C > G	Chr10	0.6119	0.8923	L/V
*CPNE1* c.2T > C	Chr13	0.7778	0.6667	M/T
*PARVA* c.893T > A ^2^	Chr15	0.6045	0.8905	V/E
*LGR6* c.887C > G	Chr16	0.5417	0.6689	S/W
*LGR6* c.571G > C	Chr16	0.5625	0.8837	A/P
*NDN* c.581T > A ^2^	Chr21	0.6045	0.8901	L/H
*PPP3CB* c.12G > C	Chr28	0.5561	0.8259	E/D
*TRIM67* c.30G > C	Chr28	0.5896	0.8901	C/W
*TRIM67* c.154G > C	Chr28	0.5896	0.8901	A/P

^1^ ΔAF_1_ and ΔAF_2_ represent absolute allele frequency differences between Pinan cattle and native cattle or foreign cattle populations, respectively (ΔAF_1_ = |AF_Pinan_ − AF_native_|, ΔAF_2_ = |AF_Pinan_ − AF_foreign_|). ^2^ Loci highlighted in blue (*NDN* c.581T > A and *PARVA* c.893T > A) were identified as having significant impacts on their encoded protein structures.

## Data Availability

The raw sequence data reported in this paper have been deposited in the Genome Sequence Archive (accession number: CRA016186) in the National Genomics Data Center, China National Center for Bioinformation/Beijing Institute of Genomics, the Chinese Academy of Sciences, which are publicly accessible at https://ngdc.cncb.ac.cn/gsa (accessed on 1 May 2024).

## Supplementary Materials

The following supporting information can be downloaded at: https://www.mdpi.com/article/10.3390/ani15111626/s1, Table S1: Information of all individuals; Table S2: Sequencing depth and alignment information for all individuals; Table S3: Positively selected genes in the Pinan cattle population; Table S4: The effects of missense positively selected loci of proteins; Figure S1: Neighbor-joining (NJ) tree of 352 individuals.
