# Supplementary figures and images for: Global Whole-Genome Resequencing of Beef Cattle Reveals Characteristic Traits Related Genes in Pinan Cattle
Source: Animals (Basel). 2025 May 31;15(11):1626. doi: 10.3390/ani15111626 (PMC12153678; doi:10.3390/ani15111626)

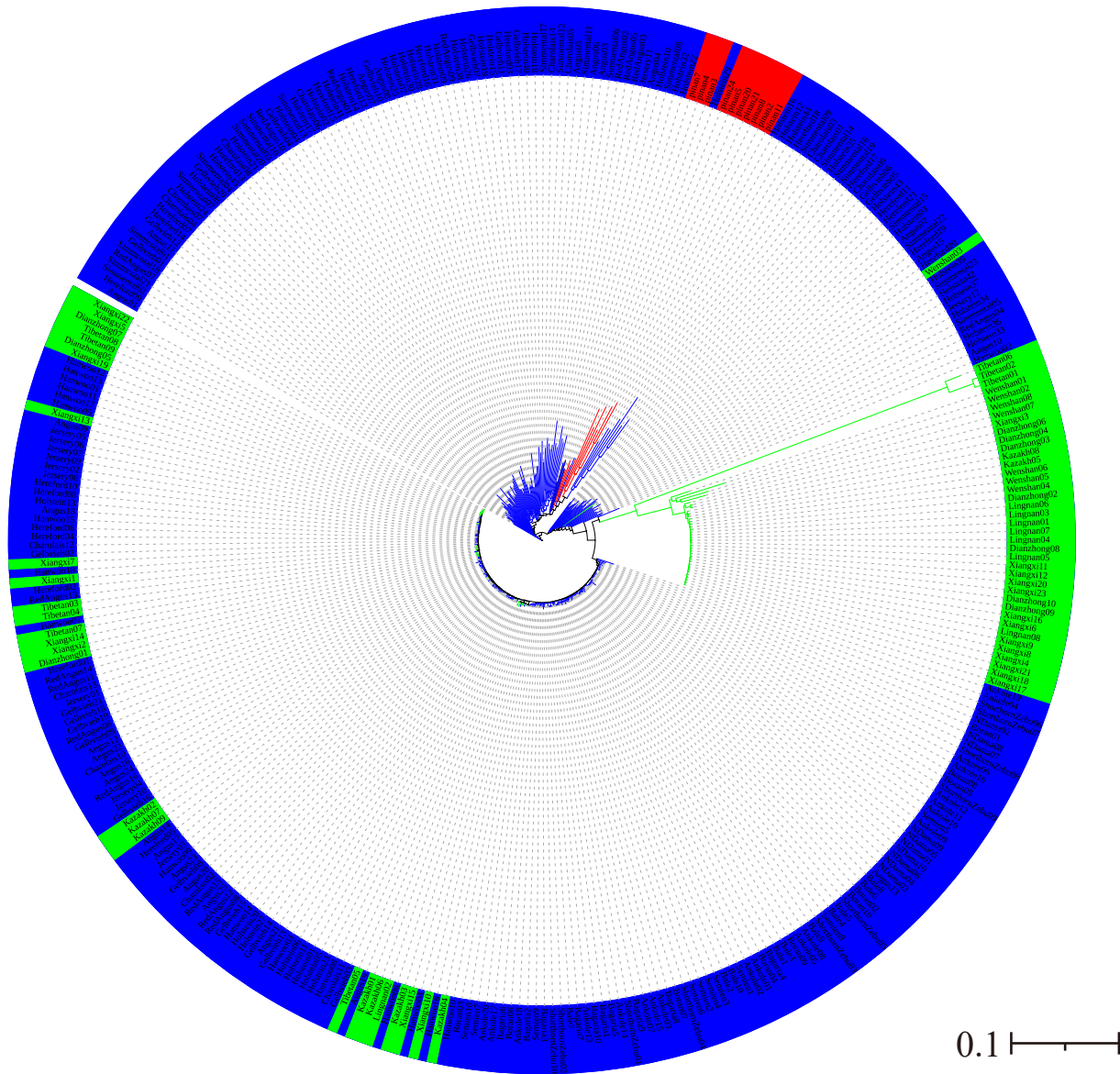

Supplement: Supplementary file 1 [file animals-15-01626-s001.zip › Supplementary Materials/FgureS1_Neighbor-joining (NJ) tree of 352 individuals..pdf]
